# Association between risk factors for injurious falls and new benzodiazepine prescribing in elderly persons

**DOI:** 10.1186/1471-2296-10-1

**Published:** 2009-01-06

**Authors:** Gillian Bartlett, Michal Abrahamowicz, Roland Grad, Marie-Pierre Sylvestre, Robyn Tamblyn

**Affiliations:** 1 Department of Family Medicine, McGill University, 515-517 Pine Avenue West, Montreal, Quebec, Canada; 2 Department of Epidemiology and Biostatistics, McGill University, 687 Pine Avenue West, V Building, Montreal, Quebec, Canada; 3 Department of Medicine, McGill University, 1140 Pine Avenue West, Montreal, Quebec, Canada

## Abstract

**Background:**

Benzodiazepines are frequently prescribed to elderly patients' despite concerns about adverse effects leading to injurious falls. Previous studies have not investigated the extent to which patients with pre-existing risk factors for falls are prescribed benzodiazepines. The objective of this study is to assess if some of the risk factors for falls are associated with new benzodiazepine prescriptions in elderly persons.

**Methods:**

Using provincial administrative databases, elderly Quebec residents were screened in 1989 for benzodiazepine use and non-users were followed for up to 5 years. Logistic regression models were used to evaluate potential predictors of new benzodiazepine use among patient baseline characteristics.

**Results:**

In the 252,811 elderly patients who had no benzodiazepine prescription during the baseline year (1989), 174,444 (69%) never filled a benzodiazepine prescription and 78,367 (31%) filled at least one benzodiazepine prescription. In the adjusted analysis, several risk factors for falls were associated with statistically significant increases in the risk of receiving a new benzodiazepine prescription including the number of prescribing physicians seen at baseline (OR: 1.12; 95% CI 1.11–1.13), being female (OR: 1.20; 95% CI 1.18–1.22) or a diagnosis of arthritis (OR: 1.11; 95% CI 1.09–1.14), depression (OR: 1.42; 95% CI 1.35–1.49) or alcohol abuse (OR: 1.24; 95% CI 1.05–1.46). The strongest predictor for starting a benzodiazepine was the use of other medications, particularly anti-depressants (OR: 1.85; 95% CI 1.75–1.95).

**Conclusion:**

Patients with pre-existing conditions that increase the risk of injurious falls are significantly more likely to receive a new prescription for a benzodiazepine. The strength of the association between previous medication use and new benzodiazepine prescriptions highlights an important medication safety issue.

## Background

Even with a growing body of evidence, controversy still surrounds the safety of benzodiazepine use especially for elderly persons. [1-7] Physiological evidence suggests that changes associated with aging make elderly people particularly sensitive to benzodiazepine side effects that have been linked with an increased risk of injuries from falls. [8-13] Despite these concerns, benzodiazepines continue to be one of the most frequently prescribed medications for elderly persons.[14] Many studies have examined the correlates of current use of benzodiazepines in elderly patients.[2,3,5,15,16] In contrast, very few have investigated predictors of new use.[6,17,18] In the studies that have looked at new benzodiazepine use, statistically significant predictors have included the presence of depressive symptoms, [18] anxiety, [4]other prescription medication use, [17] recent hospitalization, [17] female gender, [18] and older age.[6,18] However, none of these findings were consistent across the studies partly because different studies examined different potential predictors. Yet to determine the true risk attached to benzodiazepine use in elderly persons, it is important to determine if patients with pre-existing risk factors for falls are more or less likely to be given a new prescription for benzodiazepines.

The objective of this study was to assess whether elderly persons who are at greater risk for injuries from falls are also more likely to become new users of benzodiazepines.

## Methods

### Study Design and Setting

A 6 year cohort of elderly Quebec residents was assembled through provincial administrative data starting January 1, 1989. The baseline period was 1989 with follow up between January 1, 1990 and December 31, 1994. A two-stage sampling process was used to assemble the study cohort. First, a stratified random sample of 1,880 Quebec physicians was selected from the master physician file of the Quebec Health Insurance Program (RAMQ). Physicians in active practice in 1989 who graduated from Quebec or foreign medical schools, and in specialties that provide medical care for the elderly (general practitioners, medical specialists, general surgeons, psychiatrists) were eligible. Secondly, all the elderly patients that were seen by these physicians between 1989 and 1994 were identified.[19] The selected physicians saw 727,295 individuals in Quebec in those five years, which correspond to approximately 89% of the total elderly population in the province.

### Participants

In order to have complete medication records, people were excluded from the study if they were under the age of 66 at the beginning of the baseline period (n = 205,547), had temporary, non-unique health insurance number (n = 4,186); were institutionalized (n = 23,247) or died (n = 18,258) during the baseline year; were hospitalized for the entire study period (n = 3,307); moved out of the province during the baseline or first year of the study (n = 9,509), or lived in an isolated region where medication would be dispensed by nursing stations (n = 697).

To compare new users of benzodiazepines with non-users, participants were excluded if they filled a benzodiazepine prescription in the baseline year or if the first prescription in the study period was a refill (n = 209,733).[6,20] New users were dispensed at least one prescription during the study period for one of the eleven benzodiazepines in the Quebec formulary between 1990 and 1994 and available in community based pharmacies (triazolam, alprazolam, bromazepam, lorazepam, oxazepam, temazepam, nitrazepam, clonazepam, chlordiazepoxide, diazepam, and flurazepam). Participants for whom there was no benzodiazepine dispensed for the entire baseline and study period were considered non-users.

### Data Sources

Data was extracted from four population health administrative databases: patient demographics, medical services, prescription claims databases (Quebec Health Insurance Program) and the hospitalization discharge summary database (Quebec Ministry of Health and Welfare). All databases were linked through an encrypted, unique health care number for each patient. The validity of these databases has been established previously. [21-23] The patient demographic database provided age, sex, postal code and date of death. The medical services database provided the type, location (e.g. inpatient, emergency room, private office), diagnosis, treating and referring physician, and date of all services provided on a fee-for-service basis (95% of all services).[24] The prescription database provided the drug, dose, duration, prescribing physician, and date of each prescription dispensed for persons aged 65 and older. The hospitalization database provided records of all hospital discharges in Quebec including discharge diagnoses, admission and discharge dates.

### Measurement of Potential Predictors

Potential predictors of new benzodiazepine use considered in this study were limited to variables identified in at least one published study as a risk factor for injuries from falls in elderly persons.[9,25-40] For each patient, data was retrieved on patient demographics, clinical and health care use, impairments and disabilities and other medication use. The variables included in each category are detailed in Table 1.

**Table 1:** Potential predictors for new benzodiazepine use measure during the baseline year.

**Predictors**	**Details**
**Patient Demographics:**	
Age in years	Measured at baseline in years
Gender	Comparing women to men.
**Measures of health care use:**	
Prescribing Physicians	Number of distinct physicians prescribing medication in baseline year.
Hospital Discharges	Number of discharges in baseline year from acute care hospitals.
Charlson Co-morbidity Index[59]	Adapted by Deyo et al for administrative databases[60] provided baseline measure of disease severity and comorbid conditions.
**Diagnosis for impairments or disabilities associated with an increase risk for falls:**	
Previous Injuries from Falls	Any fracture or soft tissue injury including fracture of the hip, upper extremity fractures, lower extremity fractures requiring medical treatment in the baseline year.
Visual Impairment	ICD-9: 360–379
Arthritis	ICD9: 274, 710 to 725–730, 733
Stroke	ICD-9: 430–438
Depression	ICD-9: 311, 298.0, 296.2, 296.3, 309.1, 300.0–300.4
Neurological Disorders	Including dementia and Parkinson's disease: ICD-9: 290, 294, 331 to 337, 340–342, 344
Seizure Disorders	Including epilepsy: ICD-9: 345, 780.3
Osteoporosis	ICD-9: 733.0, 731.0
Misc. Impairments	any other conditions not included in other categories that could increase the risk of falls or injuries (i.e. sprained knee)
Alcohol Abuse	and/or dependence: ICD-9: 303, 291, 305.0, 535.3, 571.0–571.3, 265.2,425.5
Drug Abuse	and/or dependence: ICD-9: 304, 305.1–305.9, 292
**Non-benzodiazepine medication protective of fractures:**	
Thiazide Diuretics & Estrogen	AHF: 40:28:00; 40:28:10; 68:12:00; 68:16:00
**Non-benzodiazepine medication associated with an increase risk of falls:**	
Anti-Depressants	AHF: 28:16:04
Anti-Psychotics	AHF: 28:16:08
Sedatives	AHF: 28:24:92
Miscellaneous Psychotropics	AHF: 28:28:00 (lithium, l-tryptophan)
**Non-benzodiazepine medications known to alter motor stability:**	
Cardiac Drugs	AHF: 24:04:00
Anti-Hypertensives	AHF: 24:08:00
Vasodilators	AHF: 24:12:00
Opioid Agonists	AHF: 28:08:08
Mixed Partial Opioid Agonists/Antagonists	AHF: 28:08:12

Whether or not a patient had an injury during baseline year due to a fall was recorded using previously validated medical services and hospitalization databases.[22] The impairments and disabilities recorded were identified based on the International Classification of Diseases, 9th revision (ICD-9).[41] In addition, all remaining diagnostic codes were reviewed by two expert clinicians to capture any conditions that could increase the risk of falls or injuries and these were included under miscellaneous impairments (Table 1). Prescriptions for drugs other than benzodiazepines were retrieved from the prescription database by drug identification number within American Hospital Formulary (AHF) classes.

Depression was identified through diagnostic codes and medication use and where both the diagnosis and the use of anti-depressant medication were considered to be risk factors for falls.[35,37,39] In order to separate patients filling prescriptions for anti-depressants from those patients with a diagnosis of depression, any patient with a diagnosis for depression regardless of medication use was considered to have depression. Another variable was created to identify those patients who filled a prescription for an anti-depressant but did not have a diagnosis of depression. Therefore, this new covariate, anti-depressant medication use, represented only those people using the medication without a recorded diagnosis of depression.

### Statistical Analyses

Frequency distributions of categorical baseline characteristics were determined and the means and standard deviations (sd) were reported for continuous variables. Chi-square tests and student's t-tests were used to compare the distribution of categorical and continuous variables, respectively, between new users and non-users of benzodiazepines. Predictors of initiating benzodiazepine use were evaluated using the multivariable logistic regression analysis. Participants who did not fill a benzodiazepine prescription during the follow-up were censored at the time of death, if they moved out of the province, were admitted to a long-term institution; or the end of the study on December 31, 1994. Statistical analyses were conducted using SAS Systems 9.0 [42] and S-Plus 4.[43]

Ethics approval for the study was provided by the McGill Faculty of Medicine Institutional Review Board and the Quebec Commission for Access to Information (CAI). As all information provided to the research team was de-identified, the need for written informed consent by individuals was waived by the CAI.

## Results

The average age of the cohort at the baseline was 73.4 years (sd = 6.0) and 52% were women. Of the final study cohort of 252,811 elderly patients who had no benzodiazepine prescription during the baseline year (1989), 174,444 (69%) never filled a benzodiazepine prescription and 78,367 (31%) went on to fill a benzodiazepine prescription over the next five years.

Table 2 compares the baseline characteristics among benzodiazepine non-users and new users. All differences between groups were statistically significant (p ≤ 0.01) except for the mean number of hospital discharges, history of injuries from falls, stroke, neurological disorders and seizure disorders. Women were more likely to receive a benzodiazepine prescription (55.7% of new users versus 50.9% of non-users). Overall, new users of benzodiazepines were more likely to be taking other medications. New users had almost a two-fold increase in use of anti-depressants and sedatives as well as a higher proportion of use of cardiac medications than non-users. One of the most marked differences was for filling at least one prescription for another psychotropic medication during the baseline year (8.8% of new users versus 5.3% of non-users). Also, 41.3% of new benzodiazepine users filled at least one prescription for medications that affect motor stability compared to 35.6% of non-users. New users were also more likely to have depression and arthritis than non-users of benzodiazepines (Table 2).

**Table 2:** Frequency or means (standard deviations) of baseline (1989) characteristics among non-users and new users of benzodiazepines among Quebec elderly.

**Variable**	**Frequency or Mean (s.d.)**		**Odds Ratios and 95% Confidence Interval**^1^
		
	**Non-Users n = 174,444**	**New Users n = 78,367**	
**Patients Demographics**			
Age (in years)	73.6 (6.1)	73.0 (5.7)	0.90 (0.89–0.91)^2^
Women	50.1%	55.7%	1.20 (1.18–1.22)
**Measures of Health Care Use**			
Number of Prescribing Physicians	1.8 (1.5)	2.1 (1.6)	1.12 (1.11–1.13)
Number of Acute-Care Hospital Discharges	0.2 (0.6)	0.2 (0.5)	0.93 (0.91–0.95)
Charlson Co-morbidity Index	0.5 (1.2)	0.5 (1.2)	0.98 (0.98–0.99)
**Diagnosis for impairments or disabilities associated with an increase risk for falls:**			
Previous Injury from a Fall	4.6%	4.6%	0.93 (0.89–0.97)
Visual Impairment	21.6%	22.7%	0.98 (0.96–1.01)
Arthritis	14.2%	17.2%	1.11 (1.09–1.14)
Stroke	3.0%	2.7%	0.91 (0.86–0.96)
Depression	2.3%	3.6%	1.42 (1.35–1.49)
Neurological Disorders	2.8%	2.9%	0.95 (0.91–1.01)
Seizure Disorders	1.8%	1.8%	0.91 (0.85–0.97)
Osteoporosis	0.5%	0.5%	1.00 (0.88–1.12)
Miscellaneous Impairments	4.1%	4.6%	1.01 (0.97–1.06)
Alcohol Abuse/Dependence	0.3%	0.3%	1.24 (1.05–1.46)
Drug Abuse/Dependence	0.2%	0.2%	1.18 (0.99–1.42)
**Non-benzodiazepine medication protective of fractures:**			
Thiazide Diuretics & Estrogens	20.6%	23.3%	1.05 (1.03–1.08)
**Non-benzodiazepine medication associated with an increase risk of falls:**			
Anti-Depressants	2.1%	4.4%	1.85 (1.75–1.95)
Anti-Psychotics	1.3%	1.7%	1.11 (1.03–1.19)
Sedatives	2.6%	4.2%	1.37 (1.31–1.44)
Other Psychotropics	0.1%	0.2%	1.39 (1.14–1.71)
**Non-benzodiazepine medications known to alter motor stability:**			
Cardiac Drugs	26.1%	30.2%	1.04 (1.02–1.06)
Anti-Hypertensives	12.0%	13.9%	1.06 (1.03–1.09)
Vasodilators	12.8%	16.5%	1.21 (1.18–1.24)
Opioid Agonists	0.6%	0.8%	1.11 (1.01–1.23)
Mixed Partial Opioid Agonists/Antagonists	10.8%	12.1%	1.02 (0.99–1.05)

1 Adjusted for all other potential predictors in multivariable logistic regression model.

2 Odds ratio per 5 year increase in age.

A statistically significant (p < .0001) higher proportion of elderly patients with at least one of the risk factors based on health care use, morbidities and medication use were prescribed a benzodiazepine (32.2% of new users versus 26.1% of non-users). The last column of Table 2 summarizes the results of the multivariable logistic regression analyses. In this analysis that adjusted for all the predictors, several risk factors for falls were associated with statistically significant increases in the risk of being started on a benzodiazepine. For example, statistically significant predictors included the number of prescribing physicians seen at baseline, being female or a diagnosis of arthritis, depression or alcohol abuse (Table 2). With each additional prescribing physician seen in the baseline period, the risk of starting a benzodiazepine increased by 12% (Table 2). The strongest predictor for starting a benzodiazepine was the use of other medications, particularly anti-depressants. Several risk factors for falls were associated with decreased likelihood of being prescribed a benzodiazepine. These include older age, having a higher number of acute-care hospital stays, having an injury from a fall that required medical attention, or a diagnosis for stroke or seizure disorders (Table 2).

## Discussion

By assessing baseline characteristics of a large cohort of elderly patient who did not use benzodiazepines for at least one year, we were able to determine that many of the pre-existing risk factors for injuries from falls significantly increased the probability of initiating benzodiazepine treatment.

Although many of the more recent studies that examine the risks associated with benzodiazepine use attempt to control for medication use and health status in elderly persons, [9-12,32] few of these studies measured health care utilization. In our study, one of the important predictors of subsequent benzodiazepine use was the number of prescribing physicians in the baseline year. The interpretation of this association is not straightforward. While the estimate is adjusted for the use of many medications, and illnesses that are associated with falls, our list of predictors was not exhaustive. The number of prescribing physicians may have been an indication of other aspects of health status, not reflected by the variables included in our analyses. For example, we were unable to comprehensively capture mental health problems and these elderly patients are known to have higher rates of physician visits.[44,45] On the other hand, this predictor may also have been a measure of an individual patient's tendency to higher utilization of the health care system, independent of the patient's actual health status. Within the context of a database study, it is impossible to make this distinction but the conjecture that this association may have partly reflected a propensity to visit doctors was supported by the fact that the association persisted even after adjusting for several measures of the patient's health status. This was consistent with the previous finding that the risk of potentially inappropriate drug combinations increased with number of physicians involved in the medical management of an elderly patient.[46]

The association between the increased number of prescribing physicians and the risk of receiving potentially inappropriate prescriptions raises an important patient safety concern. [46-48] One strong trend demonstrated in our study is the increased risk of receiving a benzodiazepine *after *receiving a prescription for an anti-psychotic or psychotropic. As indicated by Avorn and Shrank in a recent publication, adverse drug reactions in elderly patients are a substantial cause of preventable illness.[49] The authors point out that an adverse drug reaction in an elderly person may be mistakenly attributed by the patient or the physician to an emerging disease or the ageing process itself due to a belief that aging is associated with inherent and inevitable disability.[49] Although many depressed elderly persons suffer from anxiety and insomnia that may be treated with a benzodiazepine, antipsychotics and other psychoactive medications may produce depression like symptoms. The subsequent use of benzodiazepines after receiving a prescription for these medications may indicate that physicians are treating a reversible adverse effect with a benzodiazepine starting a medication "cascade" rather than reducing the dose or discontinuing the problematic medication. At the very least, the fact that the previous use of these medications is associated with an increased risk of receiving a benzodiazepine may result in the exacerbation of any existing iatrogenic illness. This requires further investigation as an adverse effect is one of the few conditions in elderly patients where a complete "cure" can be expected. [44,49]

Despite the obvious lack of some details on the patients' health status, the use of administrative databases in our study offered several advantages. The main advantage was in sample size resulting in high statistical power for hypothesis testing and precision of the estimation. One of the limitations, common for pharmacoepidemiologic studies using information from administrative health databases, was that filling a prescription was only a proxy for actual benzodiazepine use.[50] Since there is evidence that physicians often prescribe benzodiazepines on an "as needed" basis and that elderly patients often show poor compliance in taking their medication, database records may overestimate the actual use of benzodiazepines.[51,52] However, this problem does not affect our results as we assess the predictors of receiving a new benzodiazepine prescription rather than the effects of the consumption of a benzodiazepine. Although our data is based on information from the early to mid 1990s, recent research indicates that benzodiazepine prescribing has not diminished, making our findings relevant to the current state of health care.[53,54]

Another problem that occurs with database studies and chart reviews, is the under-diagnosis and under-reporting of the treatment of certain diseases.[55] This likely limited our ability to examine the association between pre-existing osteoporosis and subsequent new benzodiazepine use. The low frequency of detected osteoporosis diagnoses, likely due to under-reporting, did not allow us to explore the role of this factor as a predictor. Also, certain conditions such as anxiety and insomnia were often not coded in the medical services billing database making it difficult to assess the association between these diagnoses and benzodiazepine use.[56]

Furthermore, there was no prescription information available during hospitalization. Although we adjusted our estimates by removing periods of hospitalization from the total period when a patient was at risk of receiving a benzodiazepine, the lack of this information may have accounted for the apparent protective effect of the number of acute care hospital stays in the baseline on subsequent new benzodiazepine use. Grad et al (1999) found that recent hospitalization increases the risk of new benzodiazepine use following discharge in community-dwelling elderly residents of Quebec.[17]

Our findings also have important implications for research on the adverse effects of benzodiazepine. Many of the risk factors for falls meet the classic criteria for confounders as they are statistically significantly associated with benzodiazepine use which is also a risk factor for falls.[57] This emphasizes the need to adjust for these factors in any study that evaluates adverse effects associated with benzodiazepine use.

## Conclusion

Evidence from our study does indicate that physicians are less likely to prescribe to elderly patients with certain risk factors for falls such more elderly patients, patients with a previous injury from a fall and patients diagnosed with a stroke or a seizure disorder. This concordance with evidence based recommendations is encouraging.[58] However, it is apparent from our results that benzodiazepines are a commonly used medication among elderly persons, even among those with other pre-existing conditions that strongly increase their risk of injuries from falls, particularly the use of other medications. This highlights an important medication safety issue.

## Competing interests

The authors declare that they have no competing interests.

## Authors' contributions

GB conceived the study, drafted the article and performed the initial statistical analysis. MA and RT participated in the conception of the study design, statistical analysis, interpretation and drafting of the manuscript. RG participated in the conception of the study design and editing the manuscript for clinical relevance. M-PS performed the additional statistical analysis. All authors read and approved the final manuscript.

## Pre-publication history

The pre-publication history for this paper can be accessed here:


